# Stochastic techno-economic analysis of alcohol-to-jet fuel production

**DOI:** 10.1186/s13068-017-0702-7

**Published:** 2017-01-19

**Authors:** Guolin Yao, Mark D. Staples, Robert Malina, Wallace E. Tyner

**Affiliations:** 1 Department of Agricultural Economics, Purdue University, 403 West State Street, West Lafayette, IN 47907-2056 USA; 2 Laboratory for Aviation and the Environment, Department of Aeronautics and Astronautics, Massachusetts Institute of Technology, 77 Massachusetts Avenue, Cambridge, MA 02139 USA; 3 Center for Environmental Sciences, Hasselt University, Martelarenlaan 42, 3500 Hasselt, Belgium

**Keywords:** Stochastic techno-economic analysis, Breakeven price distributions, Stochastic dominance, Aviation biofuel, Alcohol-to-jet, Monte Carlo simulation

## Abstract

**Background:**

Alcohol-to-jet (ATJ) is one of the technical feasible biofuel technologies. It produces jet fuel from sugary, starchy, and lignocellulosic biomass, such as sugarcane, corn grain, and switchgrass, via fermentation of sugars to ethanol or other alcohols. This study assesses the ATJ biofuel production pathway for these three biomass feedstocks, and advances existing techno-economic analyses of biofuels in three ways. First, we incorporate technical uncertainty for all by-products and co-products though statistical linkages between conversion efficiencies and input and output levels. Second, future price uncertainty is based on case-by-case time-series estimation, and a local sensitivity analysis is conducted with respect to each uncertain variable. Third, breakeven price distributions are developed to communicate the inherent uncertainty in breakeven price. This research also considers uncertainties in utility input requirements, fuel and by-product outputs, as well as price uncertainties for all major inputs, products, and co-products. All analyses are done from the perspective of a private firm.

**Results:**

The stochastic dominance results of net present values (NPV) and breakeven price distributions show that sugarcane is the lowest cost feedstock over the entire range of uncertainty with the least risks, followed by corn grain and switchgrass, with the mean breakeven jet fuel prices being $0.96/L ($3.65/gal), $1.01/L ($3.84/gal), and $1.38/L ($5.21/gal), respectively. The variation of revenues from by-products in corn grain pathway can significantly impact its profitability. Sensitivity analyses show that technical uncertainty significantly impacts breakeven price and NPV distributions.

**Conclusions:**

Technical uncertainty is critical in determining the economic performance of the ATJ fuel pathway. Technical uncertainty needs to be considered in future economic analyses. The variation of revenues from by-products plays a significant role in profitability. With the distribution of breakeven prices, potential investors can apply whatever risk preferences they like to determine an appropriate bid or breakeven price that matches their risk profile.

**Electronic supplementary material:**

The online version of this article (doi:10.1186/s13068-017-0702-7) contains supplementary material, which is available to authorized users.

## Background

Aviation currently accounts for approximately 5% of total anthropogenic radiative forcing [1, 2]. In the absence of mitigation measures, total greenhouse gas (GHG) emissions associated with aviation are expected to be 400–600% higher in 2050 than in 2010, driven by an increase in global air traffic of up to seven times [3]. Against this backdrop, the International Air Transport Association [4] (IATA) has set a goal of carbon-neutral growth of aviation by 2020, and a 50% reduction of CO_2_ emissions by 2050 compared to 2005 levels. Similarly, the United States (US) Federal Aviation Administration [5] (FAA) aims for carbon-neutral growth of aviation by 2020. These goals are to be achieved by improvements in aircraft operations, airport and air traffic management, airframe and engine technologies, as well as through the large-scale introduction of biofuels with significantly lower GHG emissions than petroleum-derived jet fuel, on a life cycle basis [6]. To date, no mandate exists specifically for aviation biofuel usage; however, these fuels can qualify under the Renewable Fuel Standard (RFS). Moreover, the US FAA has set a short-term goal of 1 billion gallons of alternative fuel consumption by 2018 for military and commercial applications [5].

Reduction in the climate impact of aviation may be achieved via the use of biofuels. However, unlike ground transportation which can transition to ethanol or electricity, aviation requires the use of energy dense, non-oxygenate, hydrocarbon, liquid fuels [7]. There are four major aviation biofuel technologies that are currently technically feasible: Fischer–Tropsch (F–T), hydroprocessed renewable esters and fatty acids (HEFA), sugar conversion (fermentation, thermochemical), and direct liquefaction (pyrolysis) [8]. In addition to the potential climate benefits, aviation biofuel production could help to meet the 36 million RFS targets by 2022, and could help reduce US dependence on energy imports and increase energy security [9]. More than twenty airlines have already used aviation biofuels blended with petroleum-derived jet fuel on thousands of passenger flights [10].

The existing biofuels TEA literature focuses mainly on bioethanol and biodiesel production. Recent biodiesel TEA literature focuses on vegetable oils for carbon chain attributes similar to petroleum diesel [11–13]. Other existing biofuel TEA literature emphasizes bioethanol production from lignocellulosic biomass, because lignocellulosic feedstocks have lower expected feedstock costs and avoid direct competition with food [14–19]. Generally, the TEA literature calculates breakeven prices, internal rates of return (IRR), and net present values (NPV), and uncertainty has been incorporated in a number of studies in order to estimate distributions of these values. Bauer and Hulteberg [20] developed a probability distribution for production cost using Monte Carlo simulation when evaluating a new thermochemical production process for isobutanol. Abubakar et al. [21] graphed the variations of mean NPV with the increase of the sample size. Sensitivity analyses conducted by Reyes Valle et al. [22] estimated how breakeven prices respond to ±30% uncertainty in fixed capital costs. Zhu et al. [23] used a sample size of 100 experimental cases to derive a breakeven price distribution when evaluating a Bench-scale woody biomass hydrothermal liquefaction (HTL) upgrading plant; however, their sample size is insufficient to estimate a breakeven price distribution without randomization and the authors did not consider how price projections and price uncertainties would influence the distribution results.

Very little existing TEA literature focuses specifically on aviation biofuel production, and most studies in the literature are deterministic. Atsonios et al. [24] modeled the ATJ process and evaluated five pathways of converting corn stover and wheat straw to aviation fuels deterministically. They obtained a $1.39/L breakeven price for an F–T plant, which is lower than for a mixed alcohols synthesis (MAS) plant. They concluded that the expected breakeven price of ATJ is higher, despite better performance in terms of carbon utilization and thermal efficiency, than the F–T Synthesis (FTS) route. Staples et al. [25] calculated breakeven prices of renewable middle distillate (diesel and jet) fuels from fermentation and advanced fermentation technologies, using sugarcane, corn grain, and switchgrass as feedstocks. The authors employed three scenarios and found that breakeven prices for sugarcane, corn grain, and switchgrass range from $0.61 to 2.63, $0.84 to 3.65, and $1.09 to 6.30/L of middle distillate fuel, respectively. Their analysis showed that breakeven prices are the most sensitive to feedstock type, fuel conversion efficiency, and feedstock costs. Pearlson et al. [26] estimated baseline breakeven prices for HEFA production ranging from $1.01 to 1.16/L. Maximizing jet fuel yield rather than total fuel yield in the HEFA process adds $0.07–0.08/L to the breakeven prices due to the increased hydrogen requirements and reduced middle distillate fuel yield. Similarly, Seber et al. [27] assessed the breakeven price of HEFA middle distillate fuel production from waste oils and tallow. The estimated breakeven prices were $0.88–$1.06/L for yellow grease (YG)-derived HEFA and $1.05–1.25/L for tallow-derived HEFA. The authors found that feedstock cost contributes the most to breakeven price, and that the breakeven price of middle distillate HEFA from YG and tallow was higher than petroleum-derived diesel fuel prices, but lower than the breakeven price of soybean oil HEFA. de Jong et al. [28] compared six short-term renewable jet fuel pathways by combining possible feedstocks and technologies, as well as ten greenfield, three retro-fitting, and nine co-locating strategies. Their results showed that HEFA is the most competitive pathway in the short term. However, none of the pathways can compete with petroleum-derived jet fuels on a price basis. Their analyses pointed out that conversion efficiency in fermentation is critical in determining breakeven prices. The authors examined the breakeven price and NPV variation ranges in different scenarios of investments, yields, feedstock prices, and hydrogen consumption. However, they did not estimate the distribution patterns of breakeven prices and NPV.

To the best of our knowledge, only one other TEA study for aviation biofuels incorporates stochasticity into key input and output variables: Bittner et al. [29] carried out a stochastic TEA of aviation biofuel from corn stover using a fast pyrolysis process. They investigated policies of reverse auction and capital subsidies, and found that reverse auction is more risk reducing.

This study makes three contributions to the existing biofuel TEA literature. First, most existing stochastic TEA analyses do not integrate the individual uncertain variables with other related variables in the process. We evaluate uncertainty in the conversion efficiency of two steps of the ATJ process, and then link related model variables by statistical estimation to the random draws from distributions of the conversion efficiency factors. The linked variables include capital costs, utility requirements, feedstock quantity, fuel and by-products output quantity, and natural gas costs, among others.

Second, we employ time-series price projection based on historical case-by-case patterns instead of conventional Brownian motion or mean reversion price assumption. Time-series estimation captures the uniqueness of the motion processes of each product market, based on historical prices [30].

Third, TEA studies generally translate all the uncertainties into NPV distributions and only calculate the breakeven prices for most likely cases. In this study, we derive breakeven price distributions by considering all combinations of uncertainties. This approach also permits stochastic dominance comparison and gives a guidance of benchmark investing price at each uncertainty level for private investors.

The point of departure for this research is the previous analysis by Staples et al. [25] on renewable middle distillate production via fermentation and advanced fermentation technologies. We extend this work by considering future price projections and introducing technical uncertainties in ATJ production, thereby developing a deeper and more comprehensive understanding of the ATJ pathway.

## Methods

### Pathway and feedstock descriptions

**Fig. 1:**
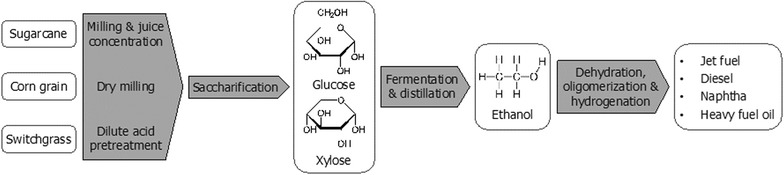
A simplified schematic of the ATJ process

ATJ involves upgrading of biomass-derived alcohols to a drop-in jet fuel or blendstock specification. Typically, ATJ technologies extract polymer sugars from a biomass feedstock via mechanical, chemical or biological means. The polymer sugars are then decomposed to monomer sugars, and metabolized (or fermented) by an engineered microorganism to an alcohol platform molecule (ethanol or isobutanol). Finally, the alcohol is dehydrated, oligomerized, and hydrogenated to a final fuel product slate which includes some proportion of drop-in jet fuel or blendstock. A number of private corporations, such as Byogy Renewables, Inc. and Gevo, Inc., has been pursuing ASTM certification and commercialization of ATJ technologies. Gevo’s ATJ production has been approved by ASTM standard in March, 2016 and up to a 30% blend in conventional jet fuel is anticipated to be used for commercial flights [31]. The subject of this analysis is a subset of ATJ technologies, which includes sugars derived from sugarcane, corn grain or switchgrass, followed by fermentation to an ethanol platform molecule. These feedstocks are selected to represent the present and future of renewable fuel production: corn grain and sugarcane are commonly used for the production of ethanol in the US and Brazil, respectively, and herbaceous lignocellulosic crops, such as switchgrass, can be used for the production of second-generation renewable fuels such as cellulosic ethanol. The final fuel product slate includes diesel, jet, heavy fuel oil, and naphtha, and we also consider non-fuel co-products from the ATJ process. ATJ derived from corn grain results in the co-production of distiller dry grains and solubles (DDGS). Bagasse produced after juice extraction from sugarcane, and biomass residues generated after sugar extraction and fermentation from switchgrass can be co-fired to meet the utility requirements of the biorefinery, and excess electricity can be exported to the grid [25]. A simplified schematic of the ATJ process is shown in Fig. 1.

### Model framework and basic assumptions

**Fig. 2:**
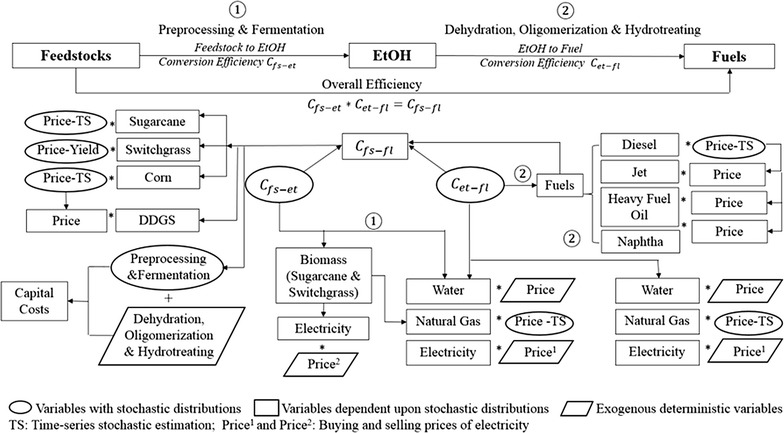
Graphical overview of technical and economic uncertainty linkages from inputs to outputs in stochastic techno-economic analysis model

Our model is designed to capture and quantify variation in ATJ process inputs, fuel outputs, and co-products. Mass and energy balance relationships, the base case scenario and the range of feasible parameter values are derived from Staples et al. [25], where the base case is defined as the most likely or mode value. We present ATJ by two main process steps: feedstock-to-ethanol conversion and ethanol-to-fuel conversion. Both steps require water, electricity, and heat (generated from natural gas) inputs. Two conversion efficiency factors are developed corresponding to the two steps, denoted as *C*_fs-et_ and *C*_et-fl_ in Fig. 2, and the product of the two conversion efficiency factors is overall conversion efficiency (see next section for more details). The two conversion efficiency factors link feedstock inputs in with fuel outputs and drive variation in the utility requirements, quantities of co-product generated, and capital costs associated with the ATJ process. *C*_fs-et_, *C*_et-fl_ and other price variables have independent stochastic distributions, represented by ovals in Fig. 2. Each iteration of the Monte Carlo simulation yields a random value from each independent stochastic distribution, and drives the changes of variables shown as rectangles in Fig. 2. Variables shown as parallelograms, such as water, power, and other inputs (enzymes, yeast, and chemicals), are less than 0.01, 0.1, and 1% of the total costs for each feedstock, respectively. Their variations do not significantly impact calculated NPV and breakeven price distributions. We treat them as exogenous and deterministic. We use @Risk, an excel add-in software, to perform Monte Carlo simulations [32].

All the price projections and breakeven price distributions are presented in real dollars. Financial analysis in this study first conducted in nominal terms and then converted to real. The deterministic assumptions in this analysis are taken from previous research by Staples et al. [25] and Seber et al. [27] assuming a facility size of 4000 bpd with 8400 operating hours per year. We assume a construction period of 3 years, followed by 20 years of production, and 8, 60, and 32% of the initial fixed capital investments are expended during the construction phase. We assume 20% equity and 80% of capital investment, financed through loans at a 5.5% interest rate for the first 10 years, and working capital is calculated as 20% of first production year (4th project year) operating costs. Since working capital is added back in the last production year, the only financial cost is the implicit interest cost of the working capital advance. We adopt the variable declining balance (VDB) depreciation method for the first 10 production years. The nominal discount rate is 15%; the income tax rate is 16.9%; and all values are presented in 2012 US dollars.

### Technical uncertainty

#### Conversion efficiency

*C*_fs-et_ and *C*_et-fl_ reflect the conversion efficiency of the feedstock-to-ethanol and ethanol-to-fuel processes, respectively. The three feedstocks considered in this analysis have different feedstock-to-ethanol conversion factors, but share the same ethanol-to-fuel conversion factor. The two conversion efficiency factors are expressed in units of kg feedstock per kg of ethanol and kg ethanol per MJ fuel, respectively, and the product of the two is the overall conversion efficiency factor in units of kg feedstock per MJ fuel.

Both the feedstock-to-ethanol and ethanol-to-fuel conversion factors are bounded and assumed to follow a PERT distribution. The PERT distribution shares the same parameters as a triangular distribution (defined by min, mode, and max values), but more of the probability density is located around the mode than a triangular distribution. The min, mode, and max values are obtained from Staples et al. [25] original technical estimation work, and the mean value of the PERT distribution is calculated as (min + 4*mode + max)/6. The min, mode, max and mean values of the low, base, and high cases are shown in Table 1.

**Table 1:** PERT distribution parameters of two conversion efficiency factors

		Min	Mode	Max	Mean
Feedstock to EtOH (kg feedstock per kg EtOH)	Corn grain	3.29	3.56	3.90	3.57
	Sugarcane	11.38	13.19	14.38	13.09
	Switchgrass	4.00	4.82	8.22	5.25
EtOH to Fuel (kg EtOH per MJ Fuel)	Corn grain				
	Sugarcane	0.03	0.04	0.07	0.04
	Switchgrass				

We assume that the total final fuel output quantities are the same for all three feedstocks, and we use statistical regressions to link the two conversion efficiency factors with feedstock inputs, utility requirements, and the share of each fuel for total fuel output. Therefore, both inputs and outputs are varied based on random draws of the two conversion efficiency factors generated in the Monte Carlo simulation.

The feedstock-to-ethanol process includes preprocessing, saccharification, and fermentation process steps. In each of these three sub-processes, the electricity, water, and heat utility requirements and output fuel shares, are correlated to the two conversion factors, *C*_fs-et_ and *C*_et-fl_, as well as the interaction between the two conversion efficiency factors. In the interaction terms, *C*_fs-et_ takes either quadratic or linear form and all of the resulting regression equations are significant with *R* ^2^ values over 0.98. The resulting equations are:

1 $${\text{input}} = \beta_{0} + \beta_{1} C_{\text{fs-et}} + \beta_{2} C_{\text{et-f;}} + \beta_{3} C_{\text{fs-et}} C_{\text{et-fl}}$$

2 $${\text{input}} = \beta_{0} + \beta_{1} C_{\text{fs-et}} + \beta_{2} C_{\text{et-f;}} + \beta_{3} C_{\text{fs-et}}^{2} C_{\text{et-fl}}$$

The ethanol-to-fuel process consists of separation and postprocessing. In each of these sub-processes, utility inputs of electricity, water, and heat, and the output fuel product shares, are determined by a quadratic function of *C*_et-fl_:

3 $${\text{input}} = \gamma_{0} + \gamma_{1} C_{\text{et-fl}} + \gamma_{2} C_{\text{et-fl}}^{2}$$

A detailed list of regressions for each utility input in each sub-process is presented in the Additional file 1: Table A1. Feedstock inputs are calculated from the input–output mass balances, and determined by the overall conversion efficiency factor. Through the three equations shown above, all input and output quantities are subject to variations in the two conversion efficiency levels.

#### Capital cost

Uncertainty in capital investment presents another aspect of technical uncertainty. Capital cost consists of two components: preprocessing and fermenter costs, and dehydration, oligomerization, and hydrotreating costs. Dehydration, oligomerization, and hydrotreating costs are treated as a linear function of facility size. Feedstock preprocessing and fermenter costs are a function of feedstock input quantity and dollars-per-unit-mass of feedstock processing capacities estimated from Staples et al. [25]. For sugarcane, the range is from $20 to 30/kg of capacity [33, 34], for corn grain the range is $55–95/kg of capacity [35, 36], and for switchgrass, the range is $115–215/kg of capacity [36, 37]. Since these capital costs are also bounded, we again choose a PERT distribution for the stochastic analysis. The modes of the preprocessing and fermenter capital cost distributions for corn grain, sugarcane, and switchgrass are $300, $347, and $697 million, respectively. The total capital cost distribution for corn grain and sugarcane follow a Beta General distribution with 90% of the values falling into the range $261–341 and $305–390 million, respectively. The total capital costs for switchgrass follows a gamma distribution with 90% of the values falling into the range from $537 to 899 million. The capital costs of preprocessing and fermenter capacity are lowest for corn grain, followed by sugarcane and switchgrass. Corn grain preprocessing is well established and is feedstock intensive; sugarcane milling involves handling the bagasse co-product; and switchgrass is a lignocellulosic process involving handling large volumes of feedstock material, as well as costly feedstock preprocessing steps.

### Price uncertainty

The future prices of the three biomass feedstocks, natural gas inputs, and diesel are projected with uncertainty. We employ two major price estimation methods: case-by-case time-series estimation is used for corn, sugarcane feedstock prices, natural gas prices, and diesel prices and contract-based price estimation, indexed by yield, is used for switchgrass prices.

#### Time-series price estimation

Future price projection is a central challenge for stochastic TEA, and in much of the literature either Brownian motion or mean reversion techniques are employed. However, neither approach is completely satisfactory: Meade [38] compared Brownian motion and mean reversion by examining daily Brent and West Texas Intermediate (WTI) crude oil prices via density forecasts. He found that Brownian motion is only accurate for one or two years, and that the addition of mean reversion does not improve the performance of the model. Postali and Picchetti [39] found that mean reversion is more accurate in representing the evolution of oil prices over time without considering structural breaks, and that geometric Brownian motion (GBM) had fewer evaluation errors with low mean reversion rate. GBM may be a better choice when no reverting trend is apparent, otherwise mean reversion is a superior choice [39]. Lucia and Schwartz [40] proposed three mean reversion models with jumps and spikes when studying energy commodity prices. He found that a price derived from a proper jump-diffusion model is closer to market price data than the GBM model in the short term. Petter and Tyner [41] found that mean reversion is a more appropriate method for diesel and gasoline price projections. From this review of the literature, there is no consistent conclusion about which method is preferred for estimating future fossil fuel prices. In addition, the motion processes underlying price movements may be different for unique commodity markets.

Given the existence of mature markets for all of the non-switchgrass inputs and outputs for the ATJ process, future prices can be projected using historical price data. Historical prices can also be used for the fuel products of the pathway, because the renewable fuels produced via the ATJ pathway have very similar performance characteristics to their petroleum-derived analogues. Therefore, we assume ATJ-derived and petroleum-derived fuels to be fungible products, up to a blend of 50% ATJ, with identical market prices. We go beyond previous analyses by employing time-series estimation using historical price data for each commodity price series, in order to forecast future feedstock, natural gas, and fuel product prices. Historical data for each commodity price are tested in order to determine the time-series process that best fits each commodity.

Corn grain and sugarcane are commodities with mature markets, and annual historical prices from 1980 to 2014 are available from the US Department of Agriculture [42, 43]. Based on Akaike information criterion (AIC), the second-order moving average (MA2) turns out to be the best price projection for corn grain and sugarcane by following the form [44]:

4 $$P_{t} = \mu + b_{1} \varepsilon_{t - 1} + b_{2} \varepsilon_{t - 2} + \varepsilon_{t},$$

where (1) *P*_*t*_ is the corn grain or sugar prices in time *t*; (2) *μ* = *E*(*P*_*t*_); (3) *ɛ*_*t*_ = *σN*_*t*_, *σ* is the volatility parameter, and $N_{t} \,\sim\,{\text{Normal}} \left( {0,1} \right)$; (4) Var(*P*_*t*_) = *σ* ^2^(1 + *b*_1_^2^ + *b*_2_^2^), and *b*_1_ and *b*_2_ are the moving average coefficients.

The upper bounds for corn grain and sugar price time-series simulation are approximately identical to their maximum historical prices, while the lower bounds sometimes generate negative values. Since negative commodity prices are unrealistic, we truncate each year’s price distribution at 0.75 times their minimum historical prices, and the fraction of the lower bound tails generated by truncation is negligible. Sugar prices are converted to sugarcane prices assuming a yield of 1 kg raw sugar from 10 kg of sugarcane [25]. All parameter estimates are presented in Table 2.

**Table 2:** Parameter estimates of time-series price projection functions

Parameters	Function type	*µ*	*σ*	*a*_1_	*b*_1_	*b*_2_	*ε*_0_	*ε*_−1_
Corn grain ($/bushel)	MA2	4.8	0.66	–	0.87	0.46	0.25	1.81
Sugar (cents/lb)	MA2	19.5	3.24	–	0.91	0.42	−1.85	1.54
Natural gas ($/thousand cubic feet)	MA1	6.9	1.3	–	0.5	–	−2.1	–
Diesel ($/gal)	ARMA11	2.72	0.44	0.94	−0.59	–	0.47	–

DDGS is a by-product of ATJ pathway from corn grain, and its prices are positively correlated with corn grain prices. It is an important revenue source in the corn grain ATJ case. We use a simple ordinary least square (OLS) regression to represent the relationship between prices of DDGS and corn grain prices with a *R* ^2^ of 0.87:

5 $${\text{Price}}\_{\text{DDGS}}_{t} = - 0.016 + 0.956*{\text{Price}}\_{\text{Corn}}_{t}$$

Natural gas accounts for over 90% of utility input costs in the base case ATJ for all three feedstocks. Natural gas is used for both heat and hydrogen production. Therefore, the variability in natural gas prices make the profitability of ATJ production more uncertain. Time-series estimation based on historical prices since 1997 is used to project future natural gas prices. AIC criterion indicates that the first-order moving average process (MA1) is the time-series stochastic projection method with the best fit, following Eq. (6), defined by the parameters shown below:

6 $$P_{t} = \mu + b_{1} \varepsilon_{t - 1} + \varepsilon_{t},$$

where (1) *P*_*t*_ is the natural gas prices in time *t*; (2) *μ* = *E*(*P*_*t*_); (3) *ɛ*_*t*_ = *σN*_*t*_, *σ* is the volatility parameter, and $N_{t} \,\sim\,{\text{Normal}} \left( {0,1} \right)$; (4) Var(*P*_*t*_) = *σ* ^2^(1 + *b*_1_^2^), and *b*_1_ is the moving average coefficient.

Similar to corn grain and sugar prices, the natural gas price distributions are truncated on the low end at 0.75 times of the minimum historical prices in order to avoid negative prices, and are converted to units of 2012 US dollars per MJ.

Sale of fuel products is the major revenue stream for the ATJ pathway. In addition to variation in the quantity of fuel produced, driven by the two conversion efficiency factors, future prices for jet, diesel, naphtha, and heavy fuel oil are also uncertain. Diesel prices are forecasted using time-series estimation, and jet, naphtha, and heavy fuel oil prices are calculated on the basis of their historical correlation with diesel prices.

Future diesel prices follow a first-order autoregressive moving average (ARMA11) process shown in Eq. (7), following the parameter estimates shown in Table 2 [45].

7 $$P_{t} - \mu = a_{1} \left( {P_{t - 1} - \mu } \right) + b_{1} \varepsilon_{t - 1} + \varepsilon_{t},$$

where (1) *P*_*t*_ is the diesel prices in time t; (2) *μ* = *E*(*P*_*t*_); (3) *ɛ*_*t*_ = *σN*_*t*_ and *σ* is the volatility parameter, and $N_{t} \,\sim\,{\text{Normal}} \left( {0,1} \right)$; (4) Var(*P*_*t*_) = *σ* ^2^(1 + *b*_1_^2^ + 2*a*_1_ *b*_1_)/(1 − *a*_1_^2^), *a*_1_ is the autoregressive coefficient, *b*_1_ is the moving average coefficient.

Historical data demonstrate that jet and diesel prices are almost identical, with correlations up to 0.996 in some periods. Ordinary least squares regression is used to regress diesel price on jet fuel, and the final regression relationship is

8 $${\text{Price}}\_{\text{Jet}}_{t} = 0.004 + 0.988*{\text{Price}}\_{\text{Diesel}}_{t}$$

Our analysis also demonstrates that heavy fuel oil and naphtha prices are highly correlated with diesel prices. We link the prices of these products to diesel prices using their historical price ratios.

#### Contract-based price estimation indexed by yield for switchgrass

In contrast to corn grain and sugarcane feedstocks, switchgrass is not currently a traded commodity, and there are no historical price data to draw upon for price forecasting. Therefore, a different approach is required for this feedstock. The cultivation of switchgrass would require farmers to make a change in their land use for a period of at least 10 years. In order to mitigate risk associated with future revenues, switchgrass producers may choose to operate under long-term price contracts [46]. Significant research exists on contract design to effectively share risk between farmers and biofuel plants. For example, Yoder et al. [46] found that contracts based on dollars-per-hectare prices, regardless of yield, were the best option for risk-averse farmers growing miscanthus, a herbaceous cellulosic crop similar to switchgrass. Therefore, this analysis assumes that switchgrass is planted and contracted using fixed dollars-per-hectare contracts.

In addition, a number of studies have estimated switchgrass yields under different production conditions. To derive our switchgrass price uncertainty ($/kg), we combine the fixed annual payment ($/ha) with varying annual yield (kg/ha) to estimate the uncertainty in unit switchgrass cost ($/kg).

The yield of switchgrass varies according to the weather conditions each year, and the ecosystem in which the crop is cultivated: switchgrass yields in upland and lowland ecosystems are reported to be distributed with mean (±standard deviation) 8.7 ± 4.2 and 12.9 ± 5.9 1000 kg/ha, respectively [47]. The coefficient of variation (CV) for upland and lowland conditions are 0.483 and 0.457, respectively. The mean of the two CVs is 0.47, the average yield for upland and lowland switchgrass is 10.8 1000 kg/ha, and we use these values to calculate the standard deviation for the average yield, which is 5.08 1000 kg/ha.

We assume the above-derived values for mean and standard deviation of switchgrass yield in order to gauge switchgrass yield uncertainty. To capture a realistic range of real world yields, we assume a bounded PERT distribution that approximates a normal distribution with the above mean and standard deviation. We set the mode to the estimated mean (10.8 1000 kg/ha) and the minimum and maximum values to ±2 standard deviations, leading to a minimum value of 0.6 1000 kg/ha and a maximum of 21.0 1000 kg/ha, respectively. The resulting mean of the PERT distribution is exactly 10.8 1000 kg/ha, with a standard deviation of 3.8 1000 kg/ha.

To derive uncertainty in unit switchgrass feedstock prices ($/kg), we combine the payment from the fixed annual farmer contract ($/ha) with varying annual yield (kg/ha). The average cost of switchgrass is estimated as $116.5/1000 kg according to a report published by the National Academy of Sciences (NAS) [48]. We use this cost together with the yield to calculate the farmer payment ($1258.2/ha):

9 $${\text{Farmer Payment}}\left( {\$ /\text{h}{\text{a}}} \right) = {\text{Switchgrass Cost }}\left( {\$ /{\text{kg}}} \right)*{\text{Mean Yields}} \left( {{\text{kg}}/\text{h}{\text{a}}} \right)$$

Using this procedure, we derive the stochastic feedstock price ($/kg) each year, which is the fixed farmer payment ($/ha) from Eq. (9) divided each year by a random draw from the switchgrass yield distribution.

Quantities of the base case for all inputs and outputs and associated prices are presented in Table 3 for an annual production of 232 million liters (61 million gallons), or approximately 4000 bpd (barrels per day), of total fuel production [25].

**Table 3:** Base case input and output quantity and price assumptions. *Source*: Staples et al. [25]

	Corn grain	Sugarcane	Switchgrass	Base prices
Water (ML)	1.39E+02	2.51E+02	1.47E+02	88.16
Power (kWh)	1.62E+08	–	–	0.07
Natural gas (MJ)	3.73E+09	6.80E+08	1.81E+09	3.88
Feedstock (kg/year)	9.75E+08	3.61E+09	1.32E+09	–
Other (enzymes, yeast, chemicals)	1.45E+07	2.09E+07	1.34E+07	1.00
DDGS (kg/year)	3.16E+8	–	–	0.17
Power for export (kWh/year)	–	4.08E+07	3.90E+07	0.05
Heavy fuel oil (L/year)	4.43E+6	4.43E+06	4.43E+06	0.65
Propane (L/year)	–	–	–	0.27
Naphtha (L/year)	1.74E+7	1.74E+07	1.74E+06	0.67
Jet (L/year)	1.96E+8	1.96E+08	1.96E+08	0.72
Diesel (L/year)	1.51E+7	1.51E+07	1.51E+07	0.72

### Breakeven jet price distributions

In addition to NPV distributions, we also develop a way to calculate and present breakeven jet price distributions. Breakeven jet price is the constant real jet price through the entire production period that makes NPV equal to zero. With the variation of the stochastic variables described previously, the diesel and jet prices that make the present value of accumulated revenues equal to the costs also changes. Breakeven price distributions permit potential investors to select any desired risk level, and then to determine the corresponding breakeven price. It also permits comparison among feedstocks.

The basic procedure is to run the standard Monte Carlo simulation and to save all the simulated values. Then the simulated values are used to calculate the breakeven price for each iteration using the Excel Goal Seek function. The breakeven prices are then fit to an appropriate standard distribution. This distribution then can be used to determine the probability for any breakeven price.

## Results and discussion

### NPV distributions

**Fig. 3:**
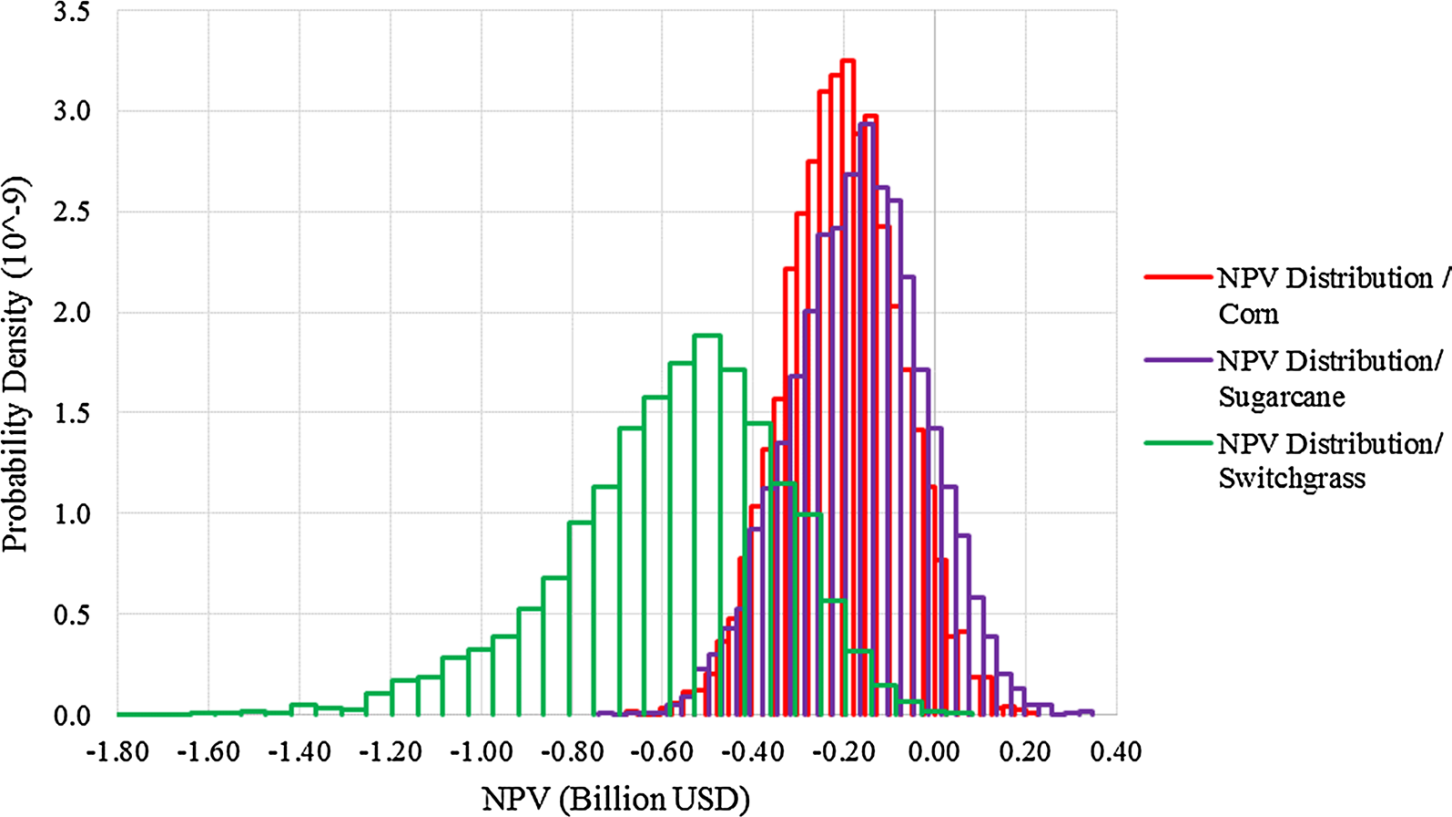
NPV probability density distributions for corn grain, sugarcane and switchgrass ATJ

A summary of NPV distribution results is presented in Table 4. The mean NPV for corn grain-, sugarcane-, and switchgrass-derived ATJ are all negative. Sugarcane has the highest NPV and smallest standard deviation, and switchgrass has the lowest NPV and largest standard deviation (Fig. 3). All three feedstocks’ probability of loss is higher than 85%, and there is more uncertainty in switchgrass ATJ fuel production. We apply stochastic dominance tests to the three distributions and found that sugarcane first-order stochastic dominates (FSD) corn and corn FSD switchgrass. The definitions of first-order and second-order stochastic dominance relationship are introduced in Additional file 1: Section A2. These results imply that under current diesel, jet and feedstock prices, technology levels, and projected future product prices, incentives would be needed to stimulate investment in aviation biofuel production via ATJ (Additional file 1: A2, Figure A1).

**Table 4:** Base case stochastic NPV distribution results for corn grain, sugarcane, and switchgrass ATJ

Statistics (Million $)	Corn grain	Sugarcane	Switchgrass
Mean	(203)	(167)	(579)
SD	123	144	239
Minimum	(610)	(829)	(1665)
Maximum	198	320	69
Probability of loss	95%	88%	100%

The NPV results show that sugarcane is the least-cost option for the ATJ pathway among the three feedstocks considered, under all circumstances. Sugarcane ATJ production does not require heat and electricity utility inputs because co-firing of the co-produced sugarcane bagasse provides more than sufficient heat and power for fuel production, and permits 168 GWh of electricity to be exported to the grid annually, in the base case. Although the combustion of biomass residues generated during switchgrass ATJ production can also offset heat and electricity requirements, it still requires more natural gas and generates less power for export than sugarcane ATJ. In addition, the conversion efficiency of switchgrass ATJ is also lower than corn grain ATJ. In general, we find that the mean NPV of the different renewable jet fuel pathways are inversely proportional to the recalcitrance of simple sugars in the raw feedstock to be converted to ethanol; switchgrass is the most recalcitrant feedstock examined (requiring greater utility, energetic and feedstock inputs per unit of monomer sugar extracted) and has the lowest NPV and, in contrast, sugarcane is the least recalcitrant feedstock (requiring fewer utility, energetic and feedstock inputs per unit of monomer sugar extracted) and has the highest mean NPV.

### Breakeven price distributions and policy implications

Fitted breakeven price distributions for corn grain-, sugarcane-, and switchgrass-derived ATJ follow normal, Beta General, and PERT distributions, respectively. The statistics and quintiles of these distributions are presented in Table 5. We find that the breakeven price distribution for switchgrass ATJ has the largest standard deviation, which is because it is represented with higher technical uncertainty than the other two processes.

**Table 5:** Fitted breakeven price distribution statistics for corn, sugarcane, and switchgrass ATJ ($/L)

Feedstocks distribution	Corn normal	Sugarcane Beta General	Switchgrass Gamma
Minimum	−∞	0.64 (2.42)	0.84 (3.17)
Maximum	∞	1.56 (5.91)	∞
Mean	1.01 (3.84)	0.97 (3.68)	1.41 (5.32)
Mode	1.01 (3.84)	0.95 (3.59)	1.32 (4.99)
Median	1.01 (3.84)	0.96 (3.65)	1.38 (5.21)
SD	0.08 (0.31)	0.12 (0.44)	0.22 (0.84)
1%	0.83 (3.13)	0.74 (2.81)	1.02 (3.85)
5%	0.88 (3.34)	0.79 (3.00)	1.10 (4.15)
15%	0.93 (3.53)	0.85 (3.21)	1.18 (4.48)
25%	0.96 (3.64)	0.89 (3.36)	1.24 (4.71)
50%	1.01 (3.84)	0.96 (3.65)	1.38 (5.21)
75%	1.07 (4.05)	1.05 (3.97)	1.53 (5.81)
95%	1.15 (4.35)	1.17 (4.44)	1.81 (6.87)
99%	1.20 (4.56)	1.25 (4.75)	1.25 (7.75)

Values in parenthesis are measured in $/gallon

**Fig. 4:**
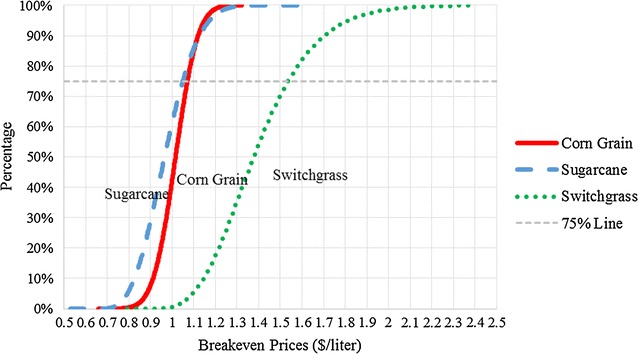
Breakeven jet price cumulative density distribution for corn grain, sugarcane, and switchgrass ATJ

The stochastic dominance relationship is presented in Fig. 4. The distribution with higher probability to have lower breakeven ATJ fuel prices is more cost efficient. By definition, switchgrass ATJ FSD corn grain and sugarcane ATJ. While we find that switchgrass-derived jet fuel first-order stochastically dominates corn and sugarcane-derived fuels, corn grain does not with regard to sugarcane, as the cumulative density functions intersect at the 90% probability level (sugarcane only second-order stochastically dominates corn). This is because DDGS prices increase with corn grain prices, which generates additional revenue when corn grain prices are high. Therefore, at higher feedstock prices, corn grain ATJ is less costly than sugarcane ATJ. However, sugarcane is the best feedstock option in ATJ fuel production under 90% of circumstances analyzed.

The mean [5 percentile; 95 percentile] breakeven jet prices per liter of ATJ from corn grain, sugarcane, and switchgrass are $1.01 [$0.88; $1.15], $0.96 [$0.79; $1.17], and $1.38 [$1.10; $1.81], respectively. The mean values are the price for jet fuel at which investors have a 50% probability of earning more than their threshold discount rate. The breakeven price distributions are within the deterministic range calculated by Staples et al. [25] confirming that our results are consistent with this analysis. Our breakeven prices for corn and sugarcane ATJ are at the lower range of breakeven prices calculated by Pearlson et al. [26] and Seber et al. [27] for HEFA pathways.

We also conduct Welch’s *t* test to determine whether the three breakeven price distributions are statistically different from each other. The two-sample test assuming unequal variances, conducted for three pairwise breakeven price samples, confirms that the mean values of the three breakeven price distributions are significantly different from each other (Additional file 1: A3, Table A2).

From a policy-perspective, risk profiles as those developed in this paper can also be used to assess the impact of alternative policies such as loan guarantees, tax credits, crop insurance, end user off-take agreements, reverse auction based on off-take contract, and capital subsidy on reducing project risk [49]. This is especially important given that de-risking investment has been shown to be one of the core levers for incentivizing a more rapid scale-up of the aviation biofuel industry [50].

### Sensitivity analysis

**Fig. 5:**
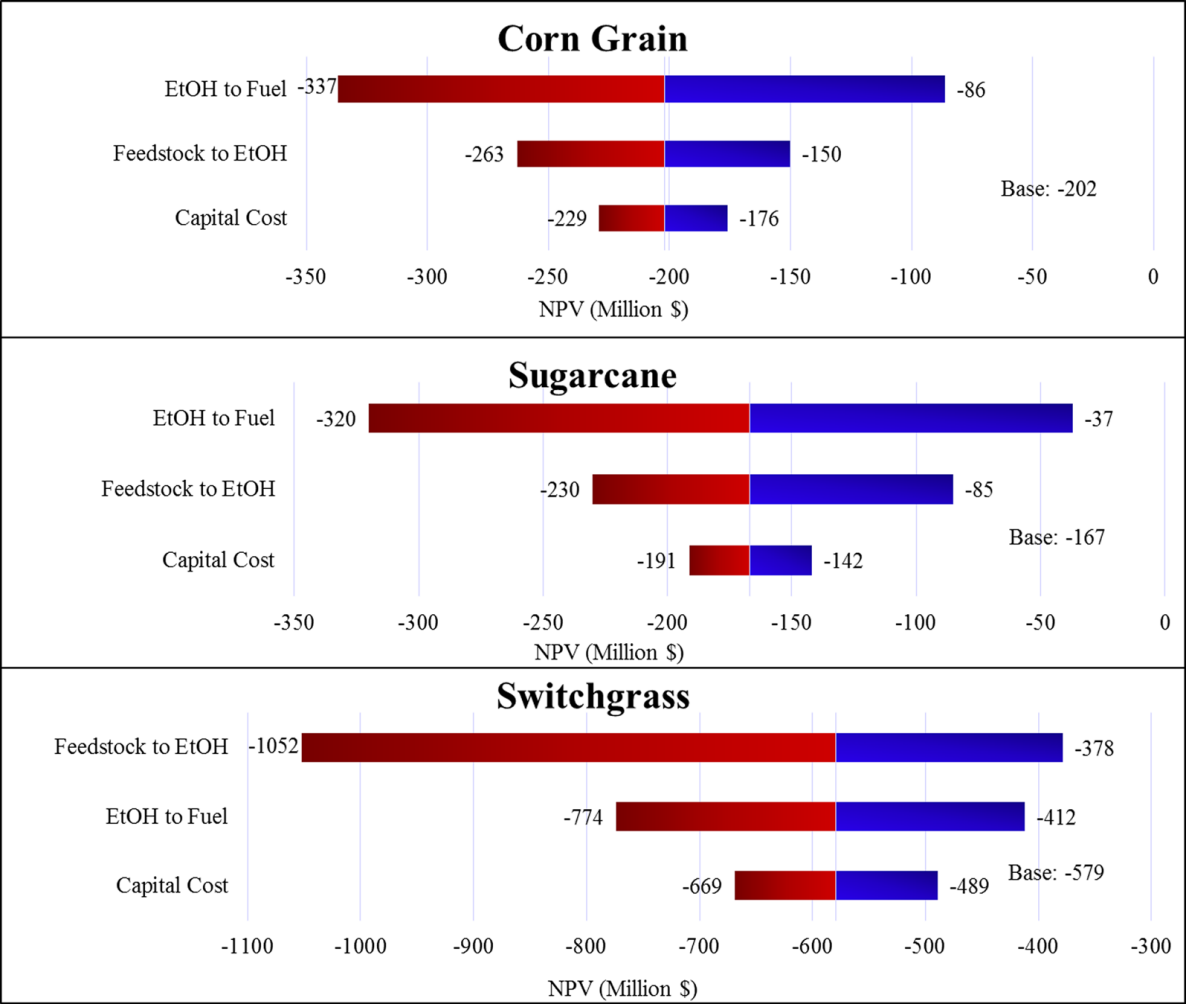
Sensitivity analyses for corn grain, sugarcane, and switchgrass ATJ

Figure 5 presents the sensitivity summary for corn grain, sugarcane, and switchgrass ATJ. The results indicate the minimum and maximum values that the NPV can achieve with variation of each individual parameter with the uncertainty ranges assumed in this analysis [51, 52]. The base case NPV is the mean value of NPV distributions with all mode input values. We only report the sensitivity results for the feedstock-to-ethanol and ethanol-to-fuel conversion factors, and the feedstock preprocessing and fermentation capital costs. Price uncertainty is not included here because there is a stochastic price variable each year for each price, which cannot be simply aggregated to a single range. The results show that the two conversion factors cause the largest impacts on NPV variation. Corn grain and sugarcane ATJ are most sensitive to ethanol-to-fuel conversion factors, followed by feedstock-to-ethanol conversion factors. In contrast, switchgrass ATJ is more sensitive to feedstock-to-ethanol conversion factors, followed by ethanol-to-fuel conversion factors. The feedstock-to-ethanol conversion factor imposes greater uncertainty for switchgrass ATJ, as compared to corn grain and sugarcane ATJ. The feedstock-to-ethanol conversion factors’ effects on corn grain and sugarcane ATJ are very similar, while its impact on switchgrass ATJ is four times larger than the impacts on corn grain and sugarcane ATJ.

## Conclusions

This study makes three contributions to current stochastic TEA: (1) we take technical uncertainty into account by linking conversion efficiency with input and output quantities through statistical methods; (2) in addition to NPV, we develop breakeven price distributions to provide potential investors the price level required to achieve their stipulated rate of return at each probability level; (3) price forecasts are based on case-by-case historical time-series analyses. Sugarcane is the lowest cost feedstock over the entire range of uncertainty with the least risks, followed by corn grain and switchgrass, with the mean breakeven jet fuel prices being $0.96/L ($3.65/gal), $1.01/L ($3.84/gal), and $1.38/L ($5.21/gal), respectively. The probability of loss given the future fuel market price projections for sugarcane, corn grain, and switchgrass ATJ are 88, 95, and 100%, respectively. Price support policies based on breakeven price distributions should be implemented to avoid potential losses and achieve targeted profitability.

Incorporating both technical and economic uncertainty is critical in characterizing the economic performance of any new technology and needs to be considered in future economic analyses. We find that the variation of revenues from by-products can impact profitability differently at different probability levels.

## Additional file

**Additional file 1.** Supplementary materials and descriptions of regression functions of each sub-process of ATJ production, first-order and second-order stochastic dominance, and Welch's t-test result for breakeven price distribution.

## Abbreviations

ATJ: alcohol-to-jet
ARMA11: first-order autoregressive moving average
DDGS: distiller dry grains and solubles
FAA: Federal Aviation Administration
F–T: Fischer–Tropsch
FTS: F–T synthesis
GBM: geometric Brownian motion
GHG: greenhouse gas
HEFA: hydroprocessed renewable esters and fatty acids
HTL: hydrothermal liquefaction
IATA: International Air Transport Association
IRR: internal rate of return
MA1: first-order moving average
MA2: second-order moving average
MAS: mixed alcohols synthesis
NPV: net present value
OLS: ordinary least squares
RFS: renewable fuel standard
TEA: techno-economic analysis
YG: yellow grease
