# Supplementary material for: Stochastic techno-economic analysis of alcohol-to-jet fuel production
Source: Biotechnol Biofuels. 2017 Jan 19;10:18. doi: 10.1186/s13068-017-0702-7 (PMC5244559; doi:10.1186/s13068-017-0702-7)
Supplement: Supplementary file 1 — Additional file 1. Supplementary materials and descriptions of regression functions of each sub-process of ATJ production, first-order and second-order stochastic dominance, and Welch's t-test result for breakeven price distribution. [file 13068_2017_702_MOESM1_ESM.docx]

**Stochastic Techno-Economic Analysis of Alcohol-to-Jet Fuel Production**

**Additional File 1**

Guolin Yao*, Mark D. Staples, Robert Malina, Wallace E. Tyner

Guolin Yao: Department of Agricultural Economics, Purdue University, 403 West State Street, West Lafayette, IN 47907-2056, USA. Email: gyao@purdue.edu.

Mark D. Staples: Laboratory for Aviation and the Environment, Department of Aeronautics and Astronautics, Massachusetts Institute of Technology, 77 Massachusetts Avenue, Cambridge, MA 02139, USA. Email: mstaples@mit.edu.

Robert Malina: Laboratory for Aviation and the Environment, Department of Aeronautics and Astronautics, Massachusetts Institute of Technology, 77 Massachusetts Avenue, Cambridge, MA 02139, USA.

Center for Environmental Sciences, Hasselt University, Martelarenlaan 42, 3500 Hasselt, Belgium. Email: robert.malina@uhasselt.be.

Wallace E. Tyner: Department of Agricultural Economics, Purdue University, 403 West State Street, West Lafayette, IN 47907-2056, USA. Email: wtyner@purdue.edu.

^*^ Corresponding Author

**A1. Regression function of each sub-process of alcohol-to-jet production**

This section briefly describes utility requirements in each sub-process of alcohol-to-jet (ATJ) production. The regression function for each sub-process that link two conversion efficiency factors C_fs-et_ and C_et-fl_ with each utility input level. Through these functions, the variation of conversion efficiency factors drives the changes in intermediate output and final output fuels.

**A1.1 A brief description of input and output flows**

ATJ production requires three basic utility inputs: electricity, heat and water. We assume that natural gas is used to produce hydrogen and heat utility. The feedstock-to-ethanol process include preprocessing, saccharification, and fermentation. The ethanol-to-fuel process consists of separation and posprocessing. Each step requires these three inputs. These basic input requirements are determined by the conversion efficiency factors through regression function. Besides input requirements, the composition shares of final fuel product slate vary based on conversion efficiency levels. We also assume that the total production quantity is constant, so the feedstock requirements change with the changes in conversion efficiency.

In the feedstock-to-ethanol process, electricity, heat, and DDGS is co-produced along with ethanol, the platform molecules. With the changes of basic input levels driven by conversion efficiency, these co-products produced will also change correspondingly. Corn grain ATJ pathway produces DDGS which generates additional revenues. Sugarcane and switchgrass ATJ pathways produce bagasse and biomass residues which can be co-fired to generate electricity and heat and re-used in production process. The changes in bagasse and biomass residue co-products drive the variations in electricity and heat demanded in both processes and further determine the natural gas demands. With variations in total output and platform molecules, the hydrogen demanded also change. In sugarcane pathway, various levels of excess electricity are exported to electric grid and generate revenues. The inclusion of these co-products can help to reduce the total costs in jet fuel production. Table A1 summarizes the regression function form of each basic utility input.

**Table A1.** Regression function forms for each utility inputs

| **Category** | **Units** | **Process** | **Sub-Procedure** | **Function Form** |
| --- | --- | --- | --- | --- |
| **Elestricity** | kWh per MJ Fuel | **Feedstock-EtOH** | **Preprocessing** | $\beta_{0}+\beta_{1}C_{fs-et}+\beta_{2}C_{et-f;}+\beta_{3}C_{fs-et}C_{et-fl}$ |
|  |  |  | **Saccharification** | $\beta_{0}+\beta_{1}C_{fs-et}+\beta_{2}C_{et-f;}+\beta_{3}C_{fs-et}^{3}C_{et-fl}$ |
|  |  |  | **Fermentation** | $\beta_{0}+\beta_{1}C_{fs-et}+\beta_{2}C_{et-f;}+\beta_{3}C_{fs-et}C_{et-fl}$ |
|  |  | **EtOH-Fuel** | **Separation** | 0 |
|  |  |  | **Postprocessing** | $\gamma_{0}+\gamma_{1}C_{et-fl}+\gamma_{2}C_{et-fl}^{2}$ |
| **Heat** | MJ NG per MJ Fuel | **Feedstock-EtOH** | **Preprocessing** | $\beta_{0}+\beta_{1}C_{fs-et}+\beta_{2}C_{et-f;}+\beta_{3}C_{fs-et}^{3}C_{et-fl}$ |
|  |  |  | **Saccharification** | 0 |
|  |  |  | **Fermentation** | 0 |
|  |  | **EtOH-Fuel** | **Separation** | 0 |
|  |  |  | **Postprocessing** | $\gamma_{0}+\gamma_{1}C_{et-fl}+\gamma_{2}C_{et-fl}^{2}$ |
|  |  |  | **Postprocessing H2** | $\gamma_{0}+\gamma_{1}C_{et-fl}+\gamma_{2}C_{et-fl}^{2}$ |
| **Water** | L water consumed per MJ Fuel | **Feedstock-EtOH** | **Preprocessing** | $\beta_{0}+\beta_{1}C_{fs-et}+\beta_{2}C_{et-f;}+\beta_{3}C_{fs-et}C_{et-fl}$ |
|  |  |  | **Saccharification** | 0 |
|  |  |  | **Fermentation** | 0 |
|  |  | **EtOH-Fuel** | **Separation** | 0 |
|  |  |  | **Postprocessing** | $\gamma_{0}+\gamma_{1}C_{et-fl}+\gamma_{2}C_{et-fl}^{2}$ |
| **Fuel Products Percentage** | MJ fuel per MJ total fuel |  | **% heavy oil** | $\gamma_{0}+\gamma_{1}C_{et-fl}+\gamma_{2}C_{et-fl}^{2}$ |
|  |  |  | **% fuel naphtha** | $\gamma_{0}+\gamma_{1}C_{et-fl}+\gamma_{2}C_{et-fl}^{2}$ |
|  |  |  | **% fuel diesel** | $\gamma_{0}+\gamma_{1}C_{et-fl}+\gamma_{2}C_{et-fl}^{2}$ |
|  |  |  | **% fuel jet** | $\gamma_{0}+\gamma_{1}C_{et-fl}+\gamma_{2}C_{et-fl}^{2}$ |

**A2. First-Order and Second-Order Stochastic Dominance [1, 2]**

Stochastic dominance offers a way to compare uncertain outcomes based on probability distributions of possible outcomes. It is built on risk-averse preferences and non-decreasing utilities.

Suppose F(•) and G(•) are two cumulative distributions defined over (-∞,∞), then:

1. F first-order stochastic dominates G (F FSD G), if F(x)≤G(x) for all x and F(x)≠G(x);
2. F second-order stochastic dominates G (F SSD G), if $\int_{-\infty}^{x} F\left( y \right)dy\leq\int_{-\infty}^{x} G\left( y \right)dy$, for all x and F(x)≠G(x).

The cumulative density distributions of corn grain, sugarcane and switchgrass ATJ are shown in Figure A1. For each value of NPV, the cumulative percentage for sugarcane is the smallest followed by corn grain and switchgrass. It means sugarcane FSD corn grain FSD switchgrass. It implies that sugarcane has higher probability to get a larger NPV. For a risk averse investor, they prefer sugarcane over corn grain over switchgrass. If F(x) ≤ G(x) for all x and F(x)≠G(x) (F FSD G), then $\int_{-\infty}^{x} F\left( y \right)dy\leq\int_{-\infty}^{x} G\left( y \right)dy$ is also valid for all x and F(x)≠G(x) (F SSD G). It means if FSD G, then F SSD G definitely, because the size of the area under F(•) up to x is smaller than that under G(•). Therefore, we can also state sugarcane SSD corn grain SSD switchgrass.


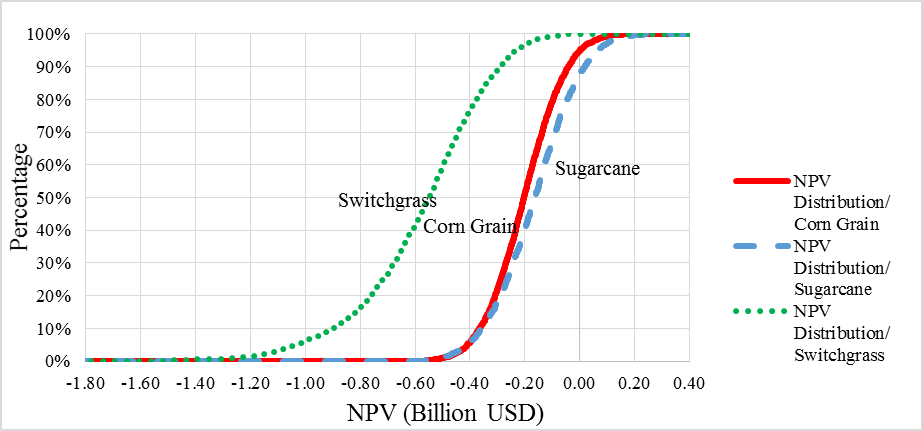


**Figure A1.** NPV cumulative density distributions for corn grain, sugarcane and switchgrass ATJ

**A3. Welch’s t-test result for breakeven price distributions**

The results of the Welch’s pairwise t-test assuming unequal variances are shown in Table A2. These results indicate that the mean breakeven price in the corn grain ATJ case is significantly higher than that of sugarcane, and lower than of the switchgrass ATJ case. The results are consistent with the mean value relationships from stochastic dominance analysis.

**Table A2.** Pairwise t-test: two-sample assuming unequal variances ($/liter)

|  | ***Corn grain*** | ***Sugarcane*** |  | ***Corn grain*** | ***Switchgrass*** |  | ***Sugarcane*** | ***Switchgrass*** |
| --- | --- | --- | --- | --- | --- | --- | --- | --- |
| **Mean** | 1.02 | 0.97 |  | 1.02 | 1.41 |  | 0.97 | 1.41 |
| **Variance** | 0.01 | 0.01 |  | 0.01 | 0.05 |  | 0.01 | 0.05 |
| **Observations** | 1000 | 1000 |  | 1000 | 1000 |  | 1000 | 1000 |
| **Hypothesized Mean Difference** | 0.00 |  |  | 0.00 |  |  | 0.00 |  |
| **df** | 1791.00 |  |  | 1262.00 |  |  | 1505.00 |  |
| **t Stat** | **9.91** |  |  | **(52.22)** |  |  | **(54.90)** |  |
| **P(T<=t) one-tail** | 0.00 |  |  | 0.00 |  |  | 0.00 |  |
| **t Critical one-tail** | 1.65 |  |  | 1.65 |  |  | 1.65 |  |
| **P(T<=t) two-tail** | 0.00 |  |  | 0.00 |  |  | 0.00 |  |
| **t Critical two-tail** | 1.96 |  |  | 1.96 |  |  | 1.96 |  |

**References**

1. Hadar J, Russell WR: **Rules for ordering uncertain prospects**. *The American Economic Review* 1969, **59**(1):25-34.

2. Mas-Colell A, Whinston MD, Green JR: **Microeconomic theory**, vol. 1: Oxford university press New York; 1995.
